# Lentviral-mediated RNAi to inhibit target gene expression of the porcine integrin αv subunit, the FMDV receptor, and against FMDV infection in PK-15 cells

**DOI:** 10.1186/1743-422X-8-428

**Published:** 2011-09-07

**Authors:** Jihuai Luo, Junzheng Du, Shandian Gao, Guofeng Zhang, Jingjing Sun, Guozheng Cong, Junjun Shao, Tong Lin, Huiyun Chang

**Affiliations:** 1 State Key Laboratory of Veterinary Etiological Biology, National Foot and Mouth Disease Reference Laboratory, Lanzhou Veterinary Research Institute, Chinese Academy of Agricultural Sciences, Lanzhou 730046, China

## Abstract

**Background:**

shRNA targeting the integrin αv subunit, which is the foot-and-mouth disease virus (FMDV) receptor, plays a key role in virus attachment to susceptible cells. We constructed a RNAi lentiviral vector, iαv pLenti6/BLOCK -iT™, which expressed siRNA targeting the FMDV receptor, the porcine integrin αv subunit, on PK-15 cells. We also produced a lentiviral stock, established an iαv-PK-15 cell line, evaluated the gene silencing efficiency of mRNA using real-time qRT-PCR, integrand αv expression by indirect immunofluorescence assay (IIF) and cell enzyme linked immunosorbent assays (cell ELISA), and investigated the in vivo inhibitory effect of shRNA on FMDV replication in PK-15 cells.

**Results:**

Our results indicated successful establishment of the iαv U6 RNAi entry vector and the iαv pLenti6/BLOCK -iT expression vector. The functional titer of obtained virus was 1.0 × 10^6 ^TU/mL. To compare with the control and mock group, the iαv-PK-15 group αv mRNA expression rate in group was reduced by 89.5%, whilst IIF and cell ELISA clearly indicated suppression in the experimental group. Thus, iαv-PK-15 cells could reduce virus growth by more than three-fold and there was a > 99% reduction in virus titer when cells were challenged with 10^2 ^TCID_50 _of FMDV.

**Conclusions:**

Iαv-PK-15 cells were demonstrated as a cell model for anti-FMDV potency testing, and this study suggests that shRNA could be a viable therapeutic approach for controlling the severity of FMD infection and spread.

## Background

Foot-and-mouth disease virus (FMDV) is a picornavirus, which causes a highly contagious disease in cloven-hoofed animals. Foot-and-mouth disease (FMD) is a threat to the worldwide livestock industry, because FMD can have a devastating effect on a country's economy. FMDV shows a high genetic and antigenic variability, which is reflected in the seven serotypes and numerous variants described to date [1]. The FMDV genome is composed of a positive-sense single-stranded RNA molecule of about 8 500 nucleotides, which contains a unique open reading frame. There are seven distinguishable serological types, *i.e*., O, A, C, Asia1, SAT1, SAT2, and SAT3, and more than 65 subtypes.

Recently, RNAi has shown promise as a therapeutic in many infections, including, viral diseases of animals and humans. RNAi is a process of sequence specific, post-transcriptional gene silencing (PTGS) in animals and plants, which is conducted using 21 to 33 nucleotides (nt) of small interfering RNA (siRNA), which are homologous in sequence to the silenced gene [2]. In eukaryotic organisms, dsRNA is produced *in vivo *or introduced by pathogens and processed into 21-23 nucleotide double-stranded short interfering RNA duplexes (siRNA) using an enzyme called Dicer, which is a member of the RNase III family of double-stranded RNA-specific endonucleases [3,4]. Each siRNA ia then incorporated into an RNA-induced silencing complex (RISC), which is an enzyme complex used to target cellular transcripts complementary to the siRNA for specific cleavage and degradation [5,6]. In addition to dsRNA, other endogenous RNA molecules have been shown to be capable of triggering gene silencing, including, short temporal RNA (stRNA) and microRNA (miRNA) [7].

RNAi has been widely studied as an antiviral technology for combating FMDV [8,9]. FMDV is antigenically variable, undergoing rapid mutation, which allows it to easily escape the host immune system via the high variability of its surface antigens. Existing vaccines and antiviral drugs have limited effectiveness, so the development of new strategies is essential. High genetic variability is a major issue that must be addressed in order to establish RNAi as a viable approach against FMDV [10]. Current measures for the control of FMDV replication include, RNAi plasmid 1D or 3D [11], VP1 [9], IRES [12], all of which are focused on several regions of the FMDV genome [13]. RNAi directed at specific gene sequences of certain FMDV strains might be a risky strategy, especially in the event of an emergent FMD outbreak in the absence of information concerning the serotype or genotype of the isolated pathogen, although early protection is needed. The integrin αv subunit is the FMDV receptor for each serotype or genotype and the integrin αv subunit of the FMDV receptor plays a key role in the attachment of the virus to susceptible cells. Studies have shown a clear correlation between sensitivity of FMDV and expression of integrins αvβ1, αvβ3, αvβ6, and αvβ8. Most integrin subunits have independent functions and are essential for normal development [14,15]. α1-null mice are viable, fertile and apparently normal, whereas α7-null mice develop muscular dystrophy, and α9-null mice die within 10 day of birth. Mutations in many of the ligands for αv integrins are also viable. Thus, although many of these ligands and αv integrins are widely expressed during development, they do not appear to be essential [16,17]. In this study, we used "knockdown" rather than "knockout" target gene expression of the porcine integrin αv subunit, the FMDV receptor, to establish a cell line that could inhibit FMDV replication. Therefore, our work on RNAi with FMDV is a step forward on all previous work, particularly with respect to the cell model system.

In this study, we constructed an RNAi lentiviral vector that inhibited target gene expression of the porcine integrin αv subunit and the FMDV receptor in PK-15, and FMDV replication in iαv-PK-15. The vector was capable of inhibiting viral replication in cultured porcine PK-15 cells.

## Methods

### Cells and viruses

293T cells and PK-15 cells were cultured in Dulbecco's modified Eagle's medium (DMEM, GIBCO™, Invitrogen Corporation, Grand Island, NY, USA) supplemented with 10% heat-inactivated fetal bovine serum (FBS) (pH 7.4). Cultures were incubated at 37°C with 5% CO_2_. FMDV O/CHA/99 [GenBank accession number AF506822] was used for viral challenge.

### Construction of Lentivirus vector and establishment of the iαv-PK-15 cell line

Double-stranded oligo [18] (ds oligo)

5'--CACCGGACGGAACAAAGACTGTTGACGAATCAACAGTCTTTGTTCCGTCC

**Figure 1:**
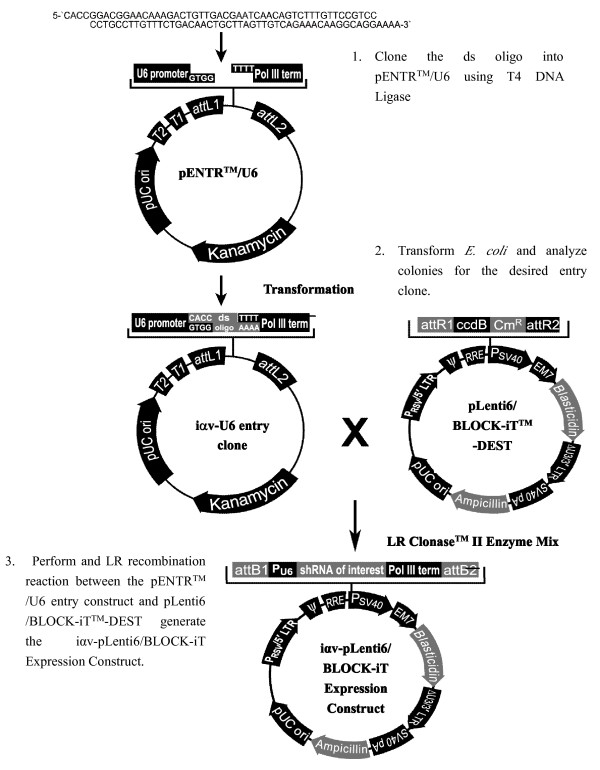
**Construction of iαv-pLenti6/BLOCK-iT Expression Construct**.

CCTGCCTTGTTTCTGACAACTGCTTAGTTGTCAGAAACAAGGCAGGAAAA--3'

was chemically designed and synthesized by the Invitrogen Corporation, and the ds oligo was cloned into the linearized pENTR™/U6 vector. The pENTR™/U6 entry vector provides a rapid and efficient way to clone oligo duplexes encoding a desired shRNA target sequence into a vector containing an RNA Pol III-dependent expression cassette for use in RNAi analysis. We generate an iαv-pLenti6/BLOCK-iT™ expression clone construct using an LR recombination reaction between the iαv-pENTR™/U6 entry clone and the pLenti6/BLOCK-iT™-DEST vector (Figure 1). We used the expression clone construct and the LR clonase enzyme mix to produce a lentiviral construct. We co-transfected the 293FT producer cell line with three optimized packaging plasmids (pLP1, pLP2, and pLP/VSVG) and the iαv-pLenti6/BLOCK-iT™ expression clone construct, which resulted in production of lentiviral stocks with a suitable titer. Stably transduced PK-15 cells were selected using blasticidin, with the minimum concentration of blasticidin required to kill untransduced PK-15 cells. The following procedure was used to determine the titer of the lentiviral stock using the PK-15 cells. To set up the stable cell line selection, we first needed to produce a lentiviral stock (iαv-PK-15 containing the packaged iαv pLenti6/BLOCK-iT™-DEST expression construct and a control containing the packaged pLenti6/BLOCK-iT™-DEST expression construct) by co-transfecting the optimized ViraPower™ packaging mix or pLenti6/BLOCK-iT™-DEST expression construct into the 293FT producer cell line. PK-15 cell cultures were incubated at 37°C with 5% CO_2_. The day before transduction, cells were trypsinized, diluted with DMEM fresh medium, and seeded into 6-well culture plates. On the day of transduction (day one), when PK-15 cells were about 80% confluent, we thawed the lentiviral stock and diluted the appropriate amount of virus (MOI = 5) into fresh DMEM complete medium. The culture medium was removed from the cells. The medium containing virus was gently mixed by pipetting and added to the cells. Polybrene was added to a final concentration of 6 μg/mL. Cells were incubated for approximately six hours prior to changing the medium. The following day (day two), the medium containing virus was removed and replaced with fresh, complete culture medium. The following day (day three), the medium was again removed and replaced with fresh, complete medium containing the appropriate amount of 2 μg/mL blasticidin to select for stably transduced cells. Selection took up to two weeks. At some point there was a massive cell death and most of the cells were washed from the bottom of the dish, leaving colonies of stable cells behind. Blasticidin-resistant colonies were picked using a Gilson pipette with a sterile tip, by lowering the tip to the surface of the colony of interest then scraping and sucking gently until they were pulled into the tip. Colonies were transferred to a well in a 24-well plate and the process was repeated with other colonies. When wells were confluent, they were split into one well of a 6-well plate (passage number 20) with a lower amount of blasticidin for maintenance (1 μg/mL). Cells were then assayed for knockdown of the target gene in one of the clones.

### Assay for knockdown of the iαv-PK-15 cells

#### RNA isolation and real-time qRT-PCR [19]

Targeted gene expression was detected by establishing iαv-PK-15 cell lines at passage number 20, which were cultured in DMEM supplemented with 10% heat inactivated FBS, and total RNA was extracted from iαv-PK-15 cell cultures using an RNeasy Mini Kit (Invitrogen), before real-time qRT-PCR analysis using the SYBP RT-PCR Kit (TaKaRa, Japan). Specific amplification was confirmed by melting curve analysis of the real-time qRT-PCR products, performed according to the manufacturer's protocol. Fluorescence was measured after each cycle and displayed graphically with a Stratagene Real time PCR Mx3000p (USA). Beta actin was used as a positive control and the αv cDNA was amplified from cDNA preparations of iαv-PK-15 cells by PCR using the primers shown in Table 1. The PCR procedures followed the manufacturer's instruction, with the following thermal profile: 10 s at 95°C for predenaturation; 40 cycles of 5 s at 95°C; and 20 s at different annealing temperatures, depending on the integrins that were amplified. The specific amplification was confirmed by melting curve analysis of the real-time qRT-PCR products according to the manufacturer's protocol, with the following thermal profile: 1 cycle of 95°C for 60 s; 55°C for 30 s; and 95°C for 30 s. PCR products were run on 3% agarose gel containing ethidium bromide and DNA bands were visualized using a UV transilluminator.

**Table 1:** Primer sequences used in this study

Primer sequence	Subunit	Size	Purpose
AF 5'-GATTGTTGTTACTGGCTGTTTTGG-3'	αv	94 bp	Real-time PCR

AR 5'-TGTTCCCTTTCCTGTTCTTCTTG-3'			

ACTF 5'-GGACTTCGAGCAGGAGATGG-3'	Β-actin	138 bp	Real-time PCR

ACTR 5'-AGGAAGGAGGGCTGGAAGAG-3'			

#### IIF

Indirect immunofluorescence (IIF) assay was carried out to detect expression of the targeted αv protein on the cell surface of an established iαv-PK-15 cell line at passage number 20, which was cultured in DMEM supplemented with 10% heat inactivated FBS. Cells were grown on acid-washed cover slips to ensure adherence of cells. The culture media was carefully aspirated from the culture dish using a pipette, because vacuum aspiration might also suck up cells from the cover slip surface. Cells were fixed in 4% paraformaldehyde (PFA) for 20 min at room temperature and were permeabilized for 5 min with 0.1% triton X-100 in 1× PBS at room temperature. Cells were then incubated in freshly prepared 50 mM NH_4_Cl for 15 min to quench free aldehyde groups from PFA, before fixation after washing three times with 1× PBS. Cultures were blocked with 1% BSA in 1× PBS for 30 minutes at room temperature, before incubation with primary antibody diluted in blocking buffer (1% BSA in PBS) for 1 h at room temperature. After primary incubation, cover slips were returned to the dishes and washed in PBS five to six times, for 5 min each wash. Secondary incubation was for 1 h at room temperature. The diluted antibody was centrifuged before use to pellet aggregates and covered in foil to protect from light. A control was included, which was comprised of the secondary antibody alone. The cover slips were returned to the dish and washed five times, for 5 min each wash using PBS, ensuring that samples were protected from the light. Cover slips were mounted by dipping the cover slips in distilled water to remove salt from PBS, before blotting the edge using filter paper to dry off excess water, before placing the cover slip cell-side down on a drop of mounting medium on a glass slide. The mounting medium used was DAPI (USA). The cover slip was stabilized on the slide using nail lacquer and left to dry overnight before viewing.

#### Indirect cell ELISA for adherent Cells

We added 100 μL of the cell suspension (1.0 × 10^5 ^cells in each well of a 96-well culture plate) and incubated in 5% CO_2 _at 37°C, for at least 24 h allowing cells to attach. We removed the residual culture medium by inversion and by gently tapping the inverted plate on a paper towel. We dispensed the primary antibody (50 μL), which was diluted in ice-cold ELISA buffer at the optimal concentration, whilst holding the pipette tip against the walls of the wells, then incubated for 1 h at 4°C. The unbound primary antibody was removed by vacuum aspiration, whilst taking care to avoid drying. The plate was washed by gently adding 200 μL of ice-cold washing buffer, whilst holding the pipette tip against the wall of the well to prevent detachment and loss of cells. The washing step was repeated four more times. After the last washing, the washing buffer was removed by inversion, whilst gently tapping the inverted plate on a paper towel. We then added the HRP-conjugated to the specific antibody for the primary antibody (50 μL) diluted in ice-cold ELISA buffer at the optimal concentration before incubating the plate for 1 h at 4°C. After five washings with PBS containing 1% BSA, 100 μL of tetramethylbenzidine (TMB) substrate was added to each well and incubation was continued for an additional 10 min at room temperature. We stopped the enzyme reaction by adding 50 μL of 1.25 M H_2_SO_4_, and the optical density (OD) was measured with an ELISA reader (iEMS-Reader; Labsystems, Helsinki, Finland) at *A*_450 nm_.

#### Viral challenge assay for the iαv-PK-15 cells

Virus infectivity was determined by serial dilution of cell samples grown in 96-well plates. The virus titer was calculated as the 50% tissue culture infective dose (TCID_50_) using the Reed-Muench formula [20]. A viral suspension titrated at 10^4.41 ^TCID_50 _was used in the experiment. Monolayers (about 50% confluent) were grown in 96-well plates to assess the capacity of viruses to grow in PK-15 cells expressing siRNAs. Cells in one well of the 96-well plates were infected with 100 TCID_50 _of virus per 0.1 ml. After 1 h of adsorption, the inoculum was removed and cells were washed twice with DMEM. The infection then proceeded in DMEM supplemented with no-fetal bovine serum and the virus titers were determined after two days. Statistical analysis was performed with the Microsoft Excel program.

## Results

### Target gene expression of the porcine integrin αv subunit (the FMDV receptor) constructed with an RNAi lentivirus vector and established as an iαv-PK-15 cell line

We constructed the iαv-pENTR™/U6 entry clone and iαv-pLenti6/BLOCK-iT™ expression clone by producing a lentiviral stock (containing the packaged iαv-pLenti6/BLOCK-iT™-DEST expression construct, or the packaged pLenti6/BLOCK-iT™-DEST expression construct) and co-transfecting the optimized ViraPower™ Packaging Mix (3.0 μg/μl) and iαv-pLenti6/BLOCK-iT™-DEST expression construct or pLenti6/BLOCK-iT™-DEST expression construct (3 μg) into the 293FT producer cell line, then harvested the viral supernatant, and determined the lentiviral stock titer with blasticidin. We picked blasticidin-resistant clones and cultured then in DMEM supplemented with 10% heat-inactivated FBS at passage number 20. The sequencing results demonstrated successful cloning of the iαv-pENTR™/U6 entry clone and the iαv-pLenti6/BLOCK -iT™ expression clone construct. The functional titer of the virus obtained was 1.0 × 10^6 ^TU/mL. We established stabled iαv-PK-15 cells line at passage number 20, which inhibited target gene expression in the porcine integrin αv subunit, the FMDV receptor.

### Real-time qRT- PCR assay of inhibition of RNA replication

**Figure 2:**
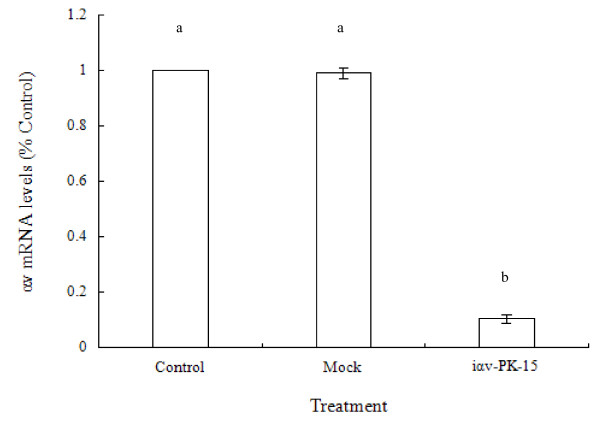
**Downregulation of αv mRNA of iαv-PK-15 detected with real-time qRT-PCR**. Relative mRNA levels of αv gene are presented as the ratio of the αv mRNA quantity and the 1 ng β-actin (ng/1 ng β-actin) to facilitate comparison with the levels of inhibition of the αv gene. Data are shown as the average of three repeated experiments. Error bars show standard deviations. There was a highly significant difference between the control and mock, and iαv-PK-15 (P < 0.01).

To demonstrate the level of inhibition, iαv-PK-15 cells were collected at passage number 20 and real-time qRT-PCR analysis was performed (Figure 2). The level of the αv gene was determined by real-time qRT-PCR and significantly decreased by about 89.5% in iαv-PK-15 cells. Out results indicate that iαv-PK-15 cells can effectively and specifically target gene expression of the porcine integrin αv subunit, the FMDV receptor.

### Level of inhibition of integrin αv subunit expression by IIF and indirect cell ELISA for adherent cells

**Figure 3:**
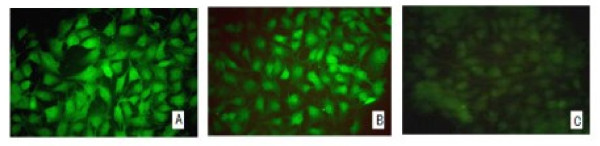
**Visualization of A. Mock (+++); B. Control (+++); and C. iαv-PK-15 (+), by IIF**. Samples were incubated with primary antibody (rabbit αv integrin, 1:500) with mouse anti-rabbit IgG-FITC (1:200). Negative (-), no fluorescent cells; weakly positive (+), the signal was faint (less than 5% of cells); positive (++), 5% to 50% were readily detected; strongly positive (+++), positive staining was intense in more than 50% of cells.

Indirect immunofluorescence was used to visualize the cells under a fluorescence microscope. Control and mock exhibited a lot of green fluorescence, whereas significant reduction was observed in the green fluorescent signal of iαv-PK-15, relative to the control and mock, indicating that iαv -PK-15 cells had inhibited expression of the surface protein (Figure 3).

**Figure 4:**
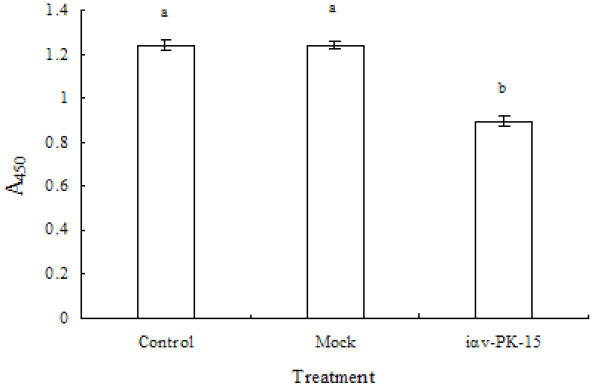
**Expression effects of integrin αv, as detected by cell ELISA method**. Cells (1.0 × 10^5^) were incubated with the isotype-matched pAb (αv integrin, 1:500) with peroxidase-conjugated mouse anti-rabbit IgG (1:10 000), and TMB. Error bars indicate standard deviations. There was a highly significant difference between the control and mock, and iαv-PK-15 (P < 0.01).

Cell ELISA was a useful technique for the quantitative analysis of cell surface antigen expression and was a sensitive, simple and reproducible method for detecting the cell membrane antigen processing machinery components of cells. Inhibition to target the gene expression of porcine integrin αv subunit, the FMDV receptor, was performed to demonstrate that the reactivity of pAb located in the membrane of cells was significantly inhibited (Figure 4).

### Inhibition of FMDV O/CHA/99 replication in iαv-PK-15 cells

**Figure 5:**
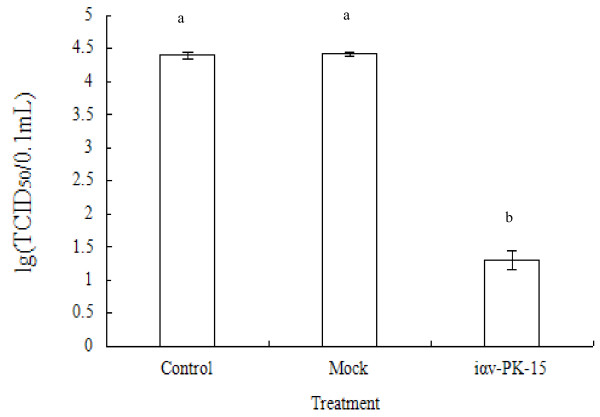
**Decrease in virus yield of iαv-PK-15 cells**. Cells (control, mock, and iαv-PK-15) were infected with 100 TCID_50 _of FMDV O/CHA/99 per 0.1 ml. The virus titer (TCID_50_) was determined three times. Error bars indicate standard deviations. There was a highly significant difference between the control and mock, and iαv-PK-15 (P < 0.01).

The antiviral activity was tested using cell monolayers in 96-well plates, where100 αL of the cell suspension (1.0 × 10^5 ^cells in each well of a 96-well culture plate) was incubated in 5% CO_2_, at 37°C, allowing at least 24 h for cells to attach. Cells were infected with 100 μl of 100 TCID_50 _FMDV O/CHA/99 and observed microscopically after 48 h. Viral infection caused a marked cytopathic effect (CPE) culminating in total cellular detachment, rounding up, and destruction, which was observed by microscopy [21]. The virus in the supernatant was titrated for infectivity using iαv-PK-15 cells. After 48 h, viral titers decreased from 10^4.39^, 10^4.41 ^in control and mock to 10^1.29 ^TCID_50 _in iαv-PK-15 cells. SiRNAs inhibited virus yield by greater than 1000-fold and there was a > 99% reduction in virus titer (Figure 5).

## Discussion

FMDV is the prototypic member of the *Aphthovirus *genus of the *Picornaviridae *family. The viral capsid is made up from 60 copies each of four viral encoded proteins, VP1 to VP4: VP1, VP2 and VP3 form the outer clasped shell; and VP4 makes up the interior surface [22]. FMDV enters the cell by receptor-mediated endocytosis, in a process that begins with the initial attachment of the virus to cell-surface receptors. The G-H loop forms a major antigenic site on the virion, which includes at its apex an Arg-Gly-Asp (RGD) motif [23,24], a sequence that has been implicated in receptor binding on the basis of synthetic peptide inhibition studies [25]. FMDV can enter cells using different pathways; the main pathway is by binding integrins via a conserved RGD located within the GH loop of VP1 [26,27]. One subgroup within the integrin family is the αv integrin, which recognizes RGD as a binding motif on natural ligands. The αv subunit is subdivided into two groups based on certain structural differences [28]. The first group is formed by α-1, α-2, α-L, α-M, and α-X. The second group is formed by α-3, α-5, α-6, α-7, α-8, α-IIb, α-V and α^IEL^. 24 functional integrins have been identified in mammalian species, which result from different pairings among 18 α subunits and 8 β subunits [29]. Different integrins have roles as FMDV receptors (αvβ1, αvβ3, αvβ6, αvβ8) [30]. Members of this group all undergo post-translational cleavage of their precursor into a heavy and a light chain. The light chain is composed of the cytoplasmic domain, the transmembrane region, and a part of the extracellular domain (about 25 kDa), whilst the heavy chain contained the rest of the extracellular domain (about 120 kDa) [31].

RNAi is a powerful gene silencing mechanism operating in most eukaryotic cells [32]. The effector molecules comprise short duplex RNA sequences of 21-23 bp that direct inhibition of homologous genes. This natural pathway is important for processing of regulatory micro RNAs (miRs) [33]. FMDV is antigenically highly variable and consists of seven serotypes and multiple subtypes [34] that underlie the rapid spreading nature of the clinical disease as early as 2-3 days post-exposure. This means that there is a need to develop new tools that provide early protection, thereby containing the disease spread. RNAi is used as an antiviral technology and has been widely studied for its affect on FMDV [9,35,36]. A multiple-siRNA expression system was developed that focused on several regions of the viral genome [37]. Focusing on the conserved regions of the viral genome [38,39], or simultaneously targeting several viral sequences are different approaches to resolving this issue. The multiple-siRNA-expressing plasmid, the pCWN11 system, is a feasible strategy to overcome the issue of high genetic variability in FMDV and viral escape [40,41]. This approach may be used to deal with several viruses, which would be especially useful in the treatment of coinfections by multiple pathogens [8]. Current measures for the control of FMD outbreak include routine vaccination, control of animal movement, and slaughter. FMD vaccines based on inactivated virus and adjuvants are effective in eliminating the disease, but risk the escape of live viruses from animal facilities or as a consequence of improper vaccine preparations [33,42]. The development of a recombinant peptide vaccine [43] and a synthetic peptide vaccine [44]) both of which are safe and effective, has been reported. The limited effectiveness of existing vaccines and antiviral drugs means the development of new strategies is essential. The RNAi approach has been reported as an ideal tool to inhibit infectious virus replication in host cells, because siRNA can target and silence important viral genes. However, RNA viruses are likely to evolve mechanisms to suppress or escape an RNAi response. Long-term silencing of viral protein expression by siRNAs has been reported to result in the emergence of viruses resistant to RNA interference [40,45]. In particular, integrin αv-heterodimers have been previously shown to mediate cellular entry of field strains of the virus. In contrast, cell surface expression of αβ3 or mRNA for the αβ1, αβ3 or αβ5 subunits did not appear to contribute to sensitivity of cells to FMDV [46]. We constructed an RNAi lentiviral vector to inhibit target gene expression of the porcine integrin αv subunit, the FMDV receptor.

It was previously shown that siRNAs inhibited virus yield by 10-fold to 1000-fold. The level of viral replication (IND 63/72) inhibition varied depending on the siRNAs produced by the Cocktail Kit. Here, the maximum inhibition (120-fold reduction in virus titer) was observed with the 2A-2C product compared with both VP3-VP1 (100-fold reduction in virus titer) and 3D-3' UTR (40-fold reduction in virus titer) at 24 h. In this case, there was a > 99% reduction in virus titer with all the serotypes up to 24 h [47]. pCWN11 was a plasmid constructed to express siRNAs with multiple-targeting of the VP1 genes in FMDV. To investigate the effect of pCWN11 on FMDV replication, approximately 10^3 ^TCID_50 _of HKN/2002 virus progeny was detected in the supernatant and collected from cells transfected with pCWN11 at 19 h p.i. Over 10^5 ^TCID_50 _of virus was determined in the supernatant collected from control cells [8]. However, a 1000-fold reduction was found in virus titer 24 h post-infection of BHK cells expressing small hairpin RNA (shRNA) under the control of a mouse U6 promoter from a plasmid construct [35]. We observed a significant cross-inhibition of FMDV replication in BHK-21 cells by siRNAs targeted at various conserved regions of the viral genome (5VNCR, VP4, VPg, POL, and 3VNCR). The results showed that siRNAs generated *in vitro *by human recombinant dicer enzyme resulted in a viral yield inhibition of 10-fold to 1000-fold in both homologous (HKN/2002) and heterologous (CHA/99) isolates of FMDV serotype O at 48 h post-infection [13]. A sequence corresponding to the critical 30 bp region that forms a stem-loop structure in IRES was also selected for investigation. The regions selected for shRNA were designated as D1, D2, D3, and D4. The virus in the supernatant was titrated in BHK-21 cells to determine infectivity. At 12, 18, and 24 h, the D4-transfected cells yielded a viral titer (10^-5 ^TCID_50_) that was almost equal to the control. The virus titer in the D2- and D3-transfected cells was less than 10^-2 ^TCID_50 _at 18 and 24 h. D1-transfected cells yielded 10^1.4^, 10^1.7^, and 10^2.9 ^TCID_50 _at 12, 18, and 24 h, respectively [48]. Kahana et al. demonstrated 100% inhibition of virus growth in BHK-21 cells transfected with a mixture of several anti-FMDV siRNAs [49]. In this study, there was reduction in virus titer of greater than 1000-fold in iαv-PK-15 cell for O/CHA/99. The results indicate that the reduction in virus titer was either better than the previous study or showed the same effect.

In this study, we constructed an RNAi lentiviral vector containing the packaged iαv-pLenti6/BLOCK-iT™-DEST expression construct to inhibit target gene expression of the porcine integrin αv subunit, the FMDV receptor. We co-transfected the ViraPower™ Packaging Mix and the iαv pLenti6/BLOCK-iT™-DEST expression construct containing the U6 RNAi cassette into 293FT cells to produce a replication-incompetent lentivirus, which was transduced into the mammalian cell line of interest. Once the lentivirus enters the target cell the viral RNA is reverse-transcribed, actively imported into the nucleus, and stably integrated into the host genome. The lentiviral construct contains a deletion in the 3' LTR that leads to self-inactivation of the lentivirus after transduction into mammalian cells. Once the lentiviral construct has integrated into the genome, the lentivirus can no longer produce packageable virus and the shRNA of interest is constitutively expressed allowing the performance of transient RNAi analysis, or blasticidin selection to generate a stable cell line for long-term knockdown studies. To determine whether this system can be used to inhibit gene expression of the integrin αv subunit gene, the expression construct and packing plasmid were transduced into PK-15 cells, and we then established an iαv-PK-15 cell line. We found that co-transduction resulted in a significant reduction in the corresponding integrin αv subunit transcription by real-time qRT-PCR, and integrand αv subunit expression by cell ELISA and IIF. Cell ELISA is a useful technique for the quantitative analysis of cell surface antigen expression, which was developed on the basis of enzyme immunohistochemistry (EIH) and ELISA. Since its development, which was facilitated by the establishment of monoclonal antibody technology, a wide range of cell types and surface molecules have been analyzed by cell ELISA, and other methods for the quantitative detection of cell surface molecules. Cell ELISA is not appropriate for analyzing mixed-cell populations. In cell ELISA, labeling of immunoreactant molecules is achieved using an enzyme, such as horseradish peroxidase (HRP). IIF is a rapid sensitive method, which uses cell culture for the detection of integrin αv subunit expression in cell surface. At present, there is no information available; further work is needed to test the relationship between anti-FMDV efficiency and integrand β1, β3, β6 and β8.

## Conclusion

Our results suggest that the interference integrin αv subunit expressing system is a feasible strategy for overcoming the issue of high genetic variability of FMDV, and viral escape. This approach may be developed to combat several viruses, which would be especially useful in the treatment of co-infections by FMDV. In addition, the new strategy would be beneficial in combating viruses with a high viral mutation rate. Our study provides a foundation for the cultivation of new varieties of transgenic organisms. However, this work represents a significant advancement, describing a new approach to trigger anti-FMDV pathways through inhibiting the FMDV receptor gene expression of the porcine integrin αv subunit.

## Competing interests

The authors declare that they have no competing interests.

## Authors' contributions

JD, SG and JL participated in planning of the study. JL carried out the majority of the experiments and drafted the manuscript. HC conceived the study and helped to draft the manuscript. GZ and JS produced the construction vector. GC, JS and TL participated in cell and virus cultures. All authors read and approved the final manuscript.
